# Toxins for Transgenic Resistance to Hemipteran Pests

**DOI:** 10.3390/toxins4060405

**Published:** 2012-06-04

**Authors:** Nanasaheb P. Chougule, Bryony C. Bonning

**Affiliations:** Department of Entomology, Iowa State University, 418 Science II, Ames, IA 50011, USA

**Keywords:** Hemiptera, aphids, plant bug, Cry toxin, plant lectins, plant protease inhibitors, transgenic plants, insect resistance

## Abstract

The sap sucking insects (Hemiptera), which include aphids, whiteflies, plant bugs and stink bugs, have emerged as major agricultural pests. The Hemiptera cause direct damage by feeding on crops, and in some cases indirect damage by transmission of plant viruses. Current management relies almost exclusively on application of classical chemical insecticides. While the development of transgenic crops expressing toxins derived from the bacterium *Bacillus thuringiensis* (Bt) has provided effective plant protection against some insect pests, Bt toxins exhibit little toxicity against sap sucking insects. Indeed, the pest status of some Hemiptera on Bt-transgenic plants has increased in the absence of pesticide application. The increased pest status of numerous hemipteran species, combined with increased prevalence of resistance to chemical insecticides, provides impetus for the development of biologically based, alternative management strategies. Here, we provide an overview of approaches toward transgenic resistance to hemipteran pests.

## 1. Hemiptera of Agricultural Importance

### 1.1. Hemiptera as Primary Pests

Insects in the order Hemiptera feed exclusively on plant sap. Most hemipterans are phytophagous and impact multiple economically important crops. Most notable of the phytophagous hemipterans are aphids, plant bugs, stink bugs, psyllids and whiteflies. Initially recognized as only minor or secondary pests, these insects have become primary pests due in part to changes in agricultural practices, such as the increased use of transgenic and classically selected plant varieties resistant to primary pests, and the decline in application of chemical insecticides. The plant bugs (*Lygus* spp.) for example have emerged as a major pest on transgenic cotton engineered to express *B thuringiensis* (Bt)-derived insecticidal toxins [1]. Hence, the hemipterans have directly benefited from the reduced application of chemical insecticides. Combined with the invasive nature and new found primary pest status, these pests negatively impact global agriculture, and currently present one of the biggest challenges for insect pest management. 

Aphids (Hemiptera: Aphididae) are exclusive phloem feeders and are among the most economically important pest insects of temperate agriculture [2]. Aphids cause major economic losses on almost all crops, and account for a large part of the 13% of agricultural output estimated to be lost to insect pests [3,4]. Economic losses result from aphid feeding, which diverts plant nutrients essential for plant growth and reproduction. The parthenogenetic reproduction of apterous (wingless) aphids allows for rapid production of high population densities under favorable conditions, while alate (winged) aphids infest new host plants [5]. In addition to the impact of feeding, aphids also transmit plant viruses; more than 275 plant viruses, nearly 50% of all insect-borne plant viruses are vectored by aphids [6,7]. Finally, aphids indirectly inhibit the photosynthetic ability of plants by producing honeydew, which allows sooty molds to grow on the leaf surface. 

Several species of plant bugs (*Lygus* spp.; Hemiptera: Miridae) are major agricultural pests, including the Western tarnished plant bug, *Lygus hesperus* Knight, the tarnished plant bug, *Lygus lineolaris* (Palisot de Beauvois), and the green plant bug, *Lygus elisus*. L*. hesperus* and *L. lineolaris* are major pests of a wide range of agronomic and horticultural crops throughout the United States and Canada [8,9,10,11]. *Lygus* spp. are reported to feed on 117 non-crop plants and over 25 cultivated plants and are primarily known as pests of cotton (*Gossypium hirsutum* L.) and seed alfalfa (*Medicago sativa* L.) [12]. Nymphs and adults feed on the flowers and fruits of many plants causing abscission and deformation [13]. Additionally, Lygus bugs have been reported as plant disease vectors [9] and may potentially transmit cotton diseases [8].

Stink bugs (Hemiptera; Pentatomidae) comprise a pest complex of critical importance, impacting 12 major crops worldwide [14]. More than 50 closely related species of stink bugs affect crops including fruit, vegetable, nut, fiber, and cereals. The most abundant and important species include the green stink bug, *Acrosternum hilare* (Say); the Southern green stink bug, *Nezara viridula* (L.); and the brown stink bug, *Euschistus servus* (Say). Stink bug losses in U.S. cotton were estimated at $64 million in 2005 and $31 million in 2008, while losses in soybean, *Glycine max* L. Merrill, were up to $13 million [15]. Other agronomic crops affected by the stink bug complex include corn, *Zea mays* L., grain sorghum, *Sorghum bicolor* L. [16], tomato, *Solanum lycopersicum* L. [17] and wheat, *Triticum aestivum* L. [18]. Additionally, the Southern green stink bug, *N viridula* L., infected with an opportunistic *Pantoea agglomerans* (Ewing and Fife) strain can transmit this pathogen causing significant damage to cotton seed as well as lint [19]. 

Whiteflies (Hemiptera: Aleyrodidae) are phloem feeders with 1500 species distributed worldwide. Several phytophagous whitefly species are major agricultural pests, including the spiraling whitefly, *Aleurodicus disperses* Russell, the greenhouse whitefly, *Trialeurodes vaporariorum* (Westwood), and the sweet potato whitefly, *Bemisia tabaci* (Gennadius) [20,21]. Whiteflies affect the biochemistry, physiology, anatomy, and development of infested plants. Similar to aphids, whiteflies feed on plant nutrients essential for plant growth and development and induce phytotoxic effects by injecting saliva into the plant [22,23]. A few species, most notably *B. tabaci*, also transmit plant-damaging viral diseases [24,25].

### 1.2. Specialization for Ingestion of Plant Sap

Hemiptera have evolved sophisticated feeding mechanisms by modification of their mouthparts into piercing-sucking structures. Their mouthparts consist of a needle-like stylet bundle with two mandibular and two maxillary stylets, and a narrow salivary canal that delivers saliva into the punctured plant tissue. The plant-feeding Hemiptera may be specialized to feed on phloem, xylem or mesophyll, or to feed on a combination of these tissues. Plant sap contains high concentrations of sugar (0.15 to 0.73 M), free amino acids (15–65 mM) and proteins. The protein content in the phloem may vary from 0.3 to 60 mg mL^−1^ according to the plant species. Plant bugs and stink bugs use extra-oral digestion by secreting copious amounts of watery saliva into the feeding site, thereby digesting sap proteins using proteases present in the saliva [26]. These insects then ingest the predigested plant nutrients for further digestion by gut proteases and for nutrient uptake. Hence, these hemipterans ingest a low volume of nutritionally complete fluid, during discrete meals or feeding bouts, relative to other hemipterans (e.g., aphids) which feed continuously and ingest large volumes of nutritionally incomplete phloem or xylem sap. These insects process large volumes of dilute plant sap and therefore must regulate osmotic pressure in the gut and hemolymph. Some continuous feeders such as the leafhoppers *Euscelidius variegatus* and* Eurymela distincta* Signoret have a filter chamber to allow excess water from the anterior midgut to bypass the midgut and move directly into the hindgut and Malpighian tubules [27,28,29]. The midgut of the pea aphid has evolved to resist the osmotic pressure generated during sap ingestion [30,31]. The anterior midgut cells contain an apical network of lamellae instead of the usual regularly arranged-microvilli. These lamellae are interlinked through 15 nm trabaculae, which increases the resistance of the tissue to stretching. 

### 1.3. Management of Hemipteran Pests

The most prevalent approach to the management of hemipteran pests is the application of classical chemical insecticides [32,33]. However, insecticides commonly lose efficacy with the development of insecticide resistance, most notably in aphids and whiteflies [34,35]. The adoption of aphid resistant crop cultivars has provided some success for the management of some species [32,36,37,38,39] and the use of natural enemies also holds promise [40,41]. 

Attempts to develop alternative tools for management of hemipteran pests including the use of Bt toxins are described below. Comprehensive information on Hemiptera-specific toxins, physiological factors contributing to the low sensitivity of Hemiptera to these toxins and potential strategies to develop more efficient insecticidal molecules are presented. 

## 2. Insecticidal Toxins Derived from *Bacillus thuringiensis*

*B. thuringenesis* (Bt) is a gram-positive, spore forming bacterium commonly found in soil. Bt produces *δ*-endotoxin insecticidal proteins (Cry and Cyt toxins) during its sporulation stage. These toxins are active against a wide range of agriculturally and medically important pests with a high degree of specificity. Delta endotoxins are pore forming toxins that may induce cell death by forming ionic pores in the membrane of midgut epithelial cells in the target insect [42,43,44,45], or by triggering the activation of a cascade signaling pathway after toxin interaction with a specific receptor in the gut membrane [46]. The mode of action of Bt toxins is complex, involves multiple steps and sequential binding to receptors and is still incompletely understood. The ingested toxin is activated by insect gut proteases, interacts with the primary receptor and then undergoes further proteolytic processing. The toxin then binds to a second receptor resulting in toxin oligomerization and insertion into the membrane forming pores that cause osmotic shock, bursting of the midgut cells and insect death [45]. 

### 2.1. Hemipteran-Active Bt Toxins

Hemipteran pests with piercing and sucking mouthparts are not particularly susceptible to the effects of Bt toxins. Low level toxicity has been reported against aphids (Table 1 and Table 2) [47,48] although the toxicity of the three Bt toxins used in this study (Cry2, Cry3 and Cry4) may have been underestimated due to the use of toxin crystals or spore suspension in feeding assays rather than presolubilized toxins. The approach used has the disadvantage that toxin solubilization would be inefficient due to the acidic pH in the aphid’s stomach [49]. Solubilized forms of four Cry δ-endotoxins (Cry1Ab, Cry3A, Cry4Aa and Cry11Aa) impacted survival of the pea aphid and retarded the growth of survivors [50]. Although these toxins showed greater aphid toxicity than previously reported, the toxicity levels were still low compared to the toxicity of some of the Cry toxins used for lepidopteran and coleopteran pest management in the field (LC50 of 1 and 3.56 μg/mL diet respectively; [51,52]). Cry3, Cry4Aa and Cry11Aa exhibited 100% mortality with ST50 values (median survival time after challenge) of less than 3.7 days at 500 µg/mL, while Cry1Ab exhibited only 25% mortality. 

**Table 1:** Toxicity of *B. thuringenesis* (Bt) toxins against aphids.

Toxin	Toxicity	Specificity	Reference
Cry2, Cry3A, Cry4	Some	Potato aphid, *Macrosiphum* *euphorbiae*	[48]
Cry4Aa Cry11Aa Cry3A,	LC50: 70-100 μg/mL 100% mortality at 500 μg/mL 60% mortality at 500 μg/mL	Pea aphid, *Acyrthosiphon pisum*	[50]
Vip1Ae-Vip2Ae	LC50: 0.576 μg/mL	Cotton aphid, *Aphis gossypii*	[53]

**Table 2:** Patented Bt toxins with toxicity against hemipteran insects.

Patent	Toxin	Specificity	Investigators
US 2009/0,068,159	TIC809, ET37, TIC810, TIC 812	*Lygus* bugs and coleopteran pests	[54]
US 2010/0,064,394	TIC853	*Lygus* bugs	[55]
US 1993/5,262,159	Bt isolates	Aphids	[47]

Vegetative insecticidal protein (Vip) purified from Bt isolates showed insecticidal activity against the cotton aphid, *Aphis gossypii* with an estimated LC50 of 0.576 µg/mL [53]. This Vip protein was identified as a binary toxin, Vip2Ae-Vip1Ae (Table 1). The recombinant, purified binary toxin bound to a 50 kDa receptor from cotton aphid brush border membrane vesicles (BBMV), but did not bind to lepidopteran gut BBMV proteins suggesting that the toxin may have aphid specificity. 

### 2.2. Bases for Low Toxicity of Bt Toxins against Sap-Sucking Insects

There are multiple factors that contribute to the low toxicity of Cry toxins against hemipteran pests. First, Bt toxins may not have evolved to kill hemipteran species as these pests are not exposed to the toxins. The bacterium *B. thuriengiensis* exists in the soil and is splashed on to the surface of foliage, and hence there is no natural selection for toxicity to Hemiptera, which pierce into the leaves, rather than feeding on the leaf surface [42]. The low susceptibility of these pests to Bt toxins may result from similarities between the glycoproteins of insect midgut microvilli, rather than as a result of direct selection for aphid toxicity [50]. 

**Figure 1:**
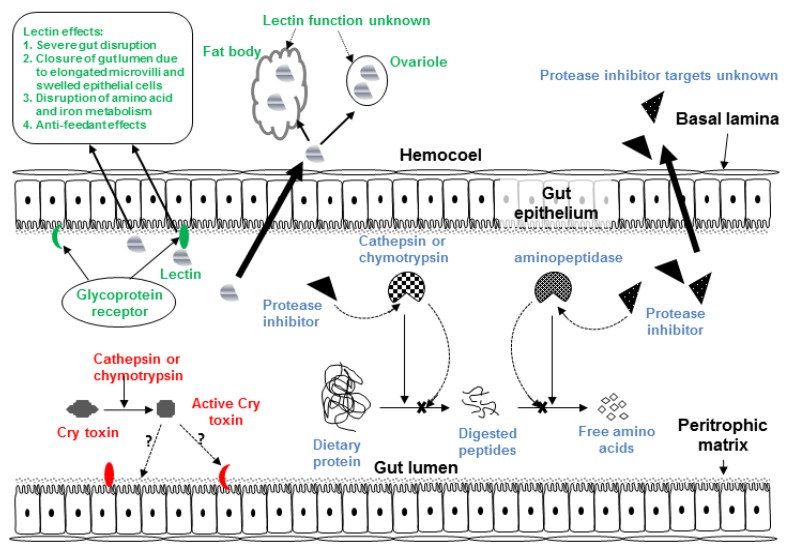
Diagram of generic insect gut and hemocoel showing target sites of Cry toxins, plant lectins and plant proteases inhibitors.

A second factor that contributes to the relatively low toxicity of Bt toxins against Hemiptera, is that proteolytic activation of the ingested Bt toxin in the insect gut is essential for toxicity. The differences in the proteolytic enzymes (type, relative abundance) and gut milieu (pH) between hemipteran and other pests are contributing factors for the low Bt toxicity against aphids [50,56]. The toxicity of Cry3A, Cry4A and Cry11A against the pea aphid was significantly increased when toxins were pre-activated with trypsin [50]. In the pea aphid gut, Bt toxins are proteolytically activated by cysteine proteases [56] (Figure 1). However, in contrast to serine proteases (trypsin and chymotrypsin) which are mostly responsible for toxin activation in the lepidopteran gut for example, most of the cysteine proteolytic activity is associated with the gut membrane, and hence potentially less accessible for degradation of gut contents. The same is true for cathepsin-L activity in the cotton aphid, *A. gossypii* [57]. Most of these agriculturally important sap-sucking pests have cysteine proteases in the gut which are active at more acidic pH relative to the alkaline conditions optimal for serine protease activity. 

In the context of Cry toxin-mediated transgenic resistance, active toxins, rather than protoxins are expressed by the transgenic plants in some cases. However, intramolecular proteolytic cleavage is also important for toxicity against insects with neutral or acidic gut pH and hence is important for hemipteran-active toxins. Intramolecular proteolytic cleavage increases the solubility of the toxin in the gut thereby facilitating acquisition. For example, chymotrypsin-treated 67 kDa Cry3A generates three polypeptides 49, 11 and 6 kDa [58] whereas trypsin or T*. molitor* gut juices generate 55, 11 and 8 kDa polypeptides. These polypeptides associate with each other and maintain insecticidal activity. Cry4Aa, which has some toxicity against the pea aphid [50], undergoes intramolecular proteolytic cleavage producing two protease-resistant fragments of 20 and 45 kDa from the 60 kDa active intermediate [59]. These two fragments associate with each other to form an active complex. A mutant resistant to intramolecular cleavage had reduced toxicity against *Culex pipiens*. 

Although functional receptors for Cry toxins have not been identified in sap-sucking pests, some studies have been carried out to investigate the association of Cry toxins with specific tissues following Cry toxin ingestion [12,56]. Immunocytochemical analysis of *L. hesperus* tissues after feeding on trypsin-activated Cry1Ac and Cry2Ab showed differential association of the toxins [12]. Cry1Ac did not associate with any of the *L. hesperus* tissues indicating the lack of a specific midgut binding receptor, whereas Cry2Ab showed extensive binding to brush border microvilli, the basement membrane of midgut epithelial cells, and to cellular structures within the hemolymph and fat body. However, Cry1Ac was associated with the pea aphid gut membrane fraction in feeding assays whereas Cry3Aa was not [56]. In competition pull down assay experiments, both toxins showed specific binding to pea aphid gut BBMV. These different results reported for Cry3Aa could result from incomplete activation of Cry3Aa in the aphid gut and instability of the toxin. In most cases toxicity is correlated with receptor binding; however there are reports of toxins binding in resistant or non-susceptible insects [60,61]. Clearly, the interactions between Cry toxins and gut receptors are complex and need further investigation at the molecular level. 

### 2.3. Cry Toxin Modification for Enhanced Hemipteran Toxicity

Despite extensive screening, relatively few toxins have been identified with significant toxicity against hemipteran pests (Table 1 and Table 2) [53]. However, there is potential for modification of Cry toxins for improved efficacy against Hemiptera. 

Proteolytic activation of Cry toxins in the insect gut is an essential step for toxicity. This step has been extensively studied using Cry1 toxins and involves the removal of 27–29 *N*-terminal amino acids and 500–600 *C*-terminal amino acids. In hemipteran pests, acquisition of active toxin appears to be a limiting step as the midgut is slightly acidic or neutral and the majority of the endoproteolytic activity is of the membrane associated cathepsin L and B type [49,62,63]. Neither of these aspects is advantageous for activation of Cry toxins. Introduction of cathepsin protease-specific cleavage sites in the Cry toxin to facilitate activation and/or intramolecular cleavage for increased solubility as appropriate, provides an ideal way to maximize toxicity. Modification of Cry3A by introducing chymotrypsin/cathepsin G sites resulted in a three-fold increase in the toxicity towards larvae of the Western corn rootworm, *Diabrotica virgifera* [64]. 

Toxin modification for improved toxin binding to the insect gut resulted in toxins with activity against a broader range of pest species, and more sustainable levels of toxicity [65]. In this toxin modification strategy, Cry1Ac was fused to the nontoxic ricin B-chain, which is a galactose/*N*-acetylgalactosamine binding lectin. This modified toxin had increased toxicity against a susceptible insect, the stem borer, *Chilo suppressalis*, as well as against a resistant insect, the cotton leaf worm, *Spodoptera littoralis*. Moreover, the modified toxin was also toxic to a hemipteran pest, the leafhopper, *Cicadulina mbila*, which is not susceptible to the native Cry1Ac. The ricin B-chain mediated Cry1Ac toxicity likely results from provision of additional toxin binding domains and consequent increased potential for interaction with gut receptor proteins. 

## 3. Plant Defense Proteins: Lectins

Lectins are carbohydrate-binding proteins that are widely distributed in animals, plants and microorganisms [66,67,68]. Non-catalytic domains of these proteins bind reversibly to specific monosaccharides or complex glycans to carry out biological functions [69]. The physiological roles of plant lectins are widely debated, ranging from growth regulation [70], plant development [71], seed storage [72] and defense against pest and pathogens [73,74]. The role of lectins in plant defense against insect herbivory is of great interest and a broad spectrum of plant lectins has been tested for insecticidal activity. 

Lectins vary widely in binding specificity, and mammalian toxicity. Indeed, lectins are present in a wide variety of plants including those that are commonly consumed such as beans, wheat, potato, and banana. Kidney bean lectin is the most well-known example of a lectin that can cause gastrointestinal stress if the beans are not processed correctly [75]. The snowdrop lectin, *Galanthus nivalis* agglutinin (GNA) is a monocot mannose-binding lectin that has received particular attention for toxicity against Hemiptera. Members of this group which include lectins from onion, leek and garlic, have no oral mammalian toxicity and are not harmful in raw or processed foods [76]. GNA is specific for the terminal α-1-3-linked mannose. The α-1-3-linked mannose residues are scarce in the brush border membranes of the mammalian small intestine. Studies using a rat model have shown that GNA is not toxic [77,78,79]. 

### 3.1. Impact of Lectins on Herbivorous Insects

The general effects of plant lectins on insect physiology such as fecundity, growth and development have been well documented [79]. Binding of plant lectins to the insect gut epithelium is a prerequisite for toxicity. Lectins appear to affect multiple insect physiological processes mediated by binding to glycoproteins on the gut membrane. GNA and ConA both bind the pea aphid gut membrane-anchored digestive enzyme aminopeptidase N (APN) [30]. APN is glycosylated with mannose, which is consistent with the binding specificities of GNA (mannose binding) and ConA (mannose and galactose binding). In contrast, wheat germ agglutinin (WGA), which has *N*-acetylglucosoamine carbohydrate specificity is not toxic to the pea aphid [80]. GNA binds to a ferritin subunit in the brown planthopper, *Nilaparvata lugens* and in *S. littoralis* causing interference with iron metabolism [81,82]. The toxicity of recombinant garlic lectin, *Allium sativum* agglutinin (ASAII), which is also insecticidal towards the pea aphid may result from interference with two physiological processes mediated by the gut membrane proteins, aminopeptidase N and sucrase [83]. The *Arum maculatum* tuber lectin ATL also bound specifically to glycoproteins in the midgut of the turnip aphid *Lipaphis erysimi* and *Aphis craccivora* resulting in insecticidal effects [84]. The jack bean lectin Concanavalin A agglutinin (Con A) binds to the entire digestive tract of the pea aphid resulting in altered amino acid metabolism and altered feeding behavior [85,86,87]. 

In addition to binding to the insect gut membrane, some GNA has been observed to cross the gut epithelium by an unknown mechanism [88]. In addition to altering midgut morphology, ingested GNA was detected in fat body, ovarioles, and hemolymph in *N. lugens* and could potentially affect multiple physiological processes at these sites [89]. 

### 3.2. Hemipteran-Active Lectins

As well as insecticidal activity against coleopteran [90,91,92,93] and lepidopteran insects [91,94], lectins are highly antinutritional and toxic to hemipterans. Lectins with specificity for mannose have the most severe effects on hemipteran pests (Table 3), although lectins with other carbohydrate specificities such as galactose, *N*-acetylglucosoamine, *N*-acetylgalactosoamine are also active against hemipteran pests such as aphids and *Lygus* bugs. GNA was the first plant lectin shown to have insecticidal activity towards aphid species and is perhaps the most studied lectin in terms of insecticidal properties. Transgenic expression of tobacco has improved resistance against the green peach aphid, *Myzus persicae*, by adversely affecting aphid populations [95,96]. Successful demonstration of the ability of GNA to provide protection against aphids led to subsequent testing of GNA for toxicity against different sap-sucking pests using a transgenic approach [97,98,99,100]. This investigation revealed that GNA is also toxic to the rice brown planthopper, *Nilaparvata lugens*, the cereal aphid, *Sitobion avenae* and the green leafhopper, *Nephotettix virescens*.

Lectins with similar binding specificity to GNA, such as the garlic lectins (*Allium sativum* agglutinins), also show toxicity against Hemiptera. *Allium sativum* produces four distinct lectins all with the same carbohydrate specificity. ASAI and ASAII are expressed in the garlic bulb while ASA-L is expressed in the leaves and ASA-R in the roots. ASA-L is toxic towards Hemiptera [101]. Transgenic tobacco and Indian mustard plants expressing ASA-L exhibited partial resistance to aphids, with reduced survival and fecundity [102,103]. Transgenic rice expressing ASA-L reduced the survival and fecundity of *N. lugens* and *N. virescens* [104,105]. These plants also showed decreased infection and replication of tungro viruses, which are transmitted by *N. lugens* and *N. virescens.* Transgenic chickpea plants expressing ASA-L under constitutive or phloem-specific promoters resulted in reduced survival and fecundity of the chickpea aphid, *A. craccivora* [106]. The mannose-binding *Pinellia ternata* agglutinin (PTA) exhibited significant insecticidal activities against hemipterans [107,108,109]. Transgenic plants expressing PTA negatively affected aphid population growth and were also toxic towards the brown planthopper. Comparative assessment of ASA-L with other GNA-like lectins (*Dieffenbachia sequina* agglutinin, DEA; *Colocasia esculenta* agglutin, CEA) for insecticidal effects against *A. craccivora*, showed that of those tested ASA-L was the most toxic [106]. Transgenic Indian mustard, *Brassica juncea*, expressing GNA, ASA-L, *Allium cepa* agglutinin (ACA, onion), and an ASA-L:ACA fusion protein negatively affected populations of the mustard aphid, *Lipaphis erysimi* with reduced survival and fecundity [110]. Survival and growth of green peach aphids, *M. persicae*, were severely affected by feeding on a diet containing the lectins GNA, *Narcissus pseudonarcissus* lectin (NPA), *A. sativum* agglutinin (ASA) or ConA [85]. 

**Table 3:** Lectins with toxicity against hemipteran pests.

Lectin (carbohydrate specificity)	Target insect toxicity (feeding assay/transgenic plant resistance)	References
GNA: *Galanthus nivalis*	Aphids (−/+)	[95,96]
agglutinin (Mannose)	Brown planthopper (+/+)	[100,111,112]
	Green peach aphid (+/−)	[85]
	Pea aphid (−/+)	[113]
	Mustard aphid (−/+)	[110]
ASA: *Allium sativum* agglutinin	Green peach aphid (+/−)	[85]
(Mannose)	Brown planthopper (−/+)	[104,105]
	Mustard aphid(−/+)	[110]
	Chickpea aphid (+/+) Tobacco aphid (−/+)	[106] [114]
ConA: Concanavalin A (Mannose, Galactose)	Green peach aphid (+/+)	[85,115]
	Pea aphid (+/−)	[116]
SNA-1: *Sambucus nigra* agglutinin(NeuAc(a-2,6)Gal/GalNAc)	Pea aphid (+/−)	[117]
WGA: Wheat germ agglutinin	Mustard aphid (+/−)	[118]
(*N*-Acetylglucosoamine)	Brown planthopper (+/+)	[111]
NPA: *Narcissus pseudonarcissus* agglutinin (Mannose)	Green peach aphid (+/−)	[85]
ATL: *Arum maculatum* tuber lectin (Mannose)	Chickpea aphid (+/−)	[106]
ACA onion: *Allium cepa* agglutinin (Galactose)	Mustard aphid (−/+)	[110]
	Pea aphid (+/+)	[116,119]
DEA: *Dieffenbachia sequina* agglutinin (thyroglobulin and asialofetuin [120]; mannose and complex sugar moities)	Chickpea aphid (+/+)	[106]
CEA: *Colocasia esculenta* agglutinin (thyroglobulin and asialofetuin [120]; mannose and complex sugar moities)	Chickpea aphid	[106]
PHA *Phaseolus vulgaris* agglutinin (Galactose, *N*-Acetylgalactosoamine)	Western tarnished plant bug;	[121]
PTA: *Pinellia ternata* agglutinin (Mannose)	Pea aphid; planthopper	[107,108,109]

The mannose and galactose binding lectin, ConA was highly toxic to pea aphid and green peach aphid [85,115,116]. Lectins with other carbohydrate specificities also exhibit toxicity against sap sucking pests in some cases. For example, the galactose-binding *Amaranthus caudatus* agglutinin (ACA) was toxic to the pea aphid and the cotton aphid, *A. gossypii* [116,119]. The *N*-acetylglucosamine binding lectin, wheat germ agglutinin (WGA) exerted negative effects on mustard aphid *L. erysimi* and *N. lugens* [111,118]. The N-acetylgalactosoamine/galactose binding lectin, phytohemagglutinin (PHA) from *Phaseous vulgaris*, binds preferentially to the midgut of the Western tarnished plant bug, *L. hesperus* Knight with deleterious consequences [121]. This study demonstrated the complex subcellular effects of PHA including severe disruption and elongation of the striated border microvilli and swelling of the epithelial cells which caused complete closure of the gut lumen. The fungal lectin, *Sclerotinia sclerotiorum* agglutinin (SSA), is toxic to the pea aphid with mortality resulting from lectin binding to the brush border membrane of the gut and induction of a signal transduction cascade leading to the death of midgut epithelial cells [122]. 

Endophytic fungi can effectively deliver lectins in plants to protect against sap-sucking pests [123]. Colonization of oilseed rape with a recombinant endophyte, *Chaetomium globosum* YY-11, expressing *Pinellia ternate* agglutinin (PTA) provided resistance against the green peach aphid. 

## 4. Antimetabolic Plant Protease Inhibitors

Plant protease inhibitors (PIs) occur naturally in a wide range of plants as a part of their natural defense system against herbivores [124,125]. Through binding to digestive proteases of phytophagous insects, PIs impair digestion [126] and suppress growth and development of herbivores [124,127]. Research on PIs has been focused on identification of PIs effective against lepidopteran and coleopteran digestive proteases and insect gut protease-mediated adaptation to PIs [128,129,130,131]. However, transgenic plants expressing PIs had limited efficacy against the targeted lepidopteran and coleopteran pests, due to the ability of these insects to adapt by downregulating expression of the targeted proteases, and upregulating expression of gut proteases that were not susceptible to the PI. 

In contrast, the use of PIs against sap feeding hemipteran pests has received limited attention. This relative lack of attention may result from the earlier premise that hemipteran insects lack proteolytic enzymes in the digestive tract, and instead rely on free amino acids in the phloem and xylem for their nutritional requirements. Inhibition of gut enzymes, including enyzmes other than proteases, is a potential area for development of transgenic resistance against hemipteran pests. 

### 4.1. The Digestive and Salivary Proteases of the Hemiptera

The importance of proteolytic digestion in agriculturally important hemipteran species has only recently been recognized with the discovery of gut digestive proteolytic activity and a large number of genes coding for proteases [132]. The biochemical characterization of proteinase activity in the guts of the pea aphid, *A. pisum* and the green peach aphid, *M. persciae* resulted in identification of significant cysteine and aminopeptidase activities (Table 4) [49,116,133,134]. These gut enzymes are membrane associated to prevent being washed away by the large volumes of ingested phloem sap. The closely related aphid species, the cereal aphid, *S. avenue* also has cysteine and chymotrypsin-like proteolytic activity in the gut [135]*.* Cathepsin-L like proteolytic activity proposed to function in the processing of exogenous ingested polypeptides was detected from the cotton aphid, *A. gossypii* [57]. The genome sequence for the pea aphid contains a large family of genes encoding cathepsin B-like proteinases [132,136]. At least five of these proteins were highly expressed in the gut.

**Table 4:** Hemipteran gut proteases and protease inhibitors with hemipteran toxicity.

Hemipteran pest	Proteolytic activity	Plant Protease inhibitors	References
Pea aphid	Cysteine and aminopeptidase	OC-I, Barley cystatin, Bowman-Birk, Serpin	[49,132,133,134,137,138,139,140]
Green peach aphid	Cysteine and aminopeptidase	OC-I	[116,138]
Cotton aphid	Cathepsin-L like	OC-I	[57,138]
Cereal aphid	Cysteine and chymotrypsin-like		[135,141]
Plant bug, *Lygus hesperus*	Aspartic and serine		[62]
Plant bug, *Lygus lineolaris*	Serine and cysteine trypsin-like and chymotrypsin-like		[26]
Planthopper	Trypsin and cathepsin B-like		[142]

Aspartic and serine proteolytic activity was detected in the gut of *L. hesperus* Knight, a major pest of cotton [62] and these enzymes were shown to be involved in digestion of dietary green fluorescent protein and casein [63]. The midgut associated serine proteolytic activity was resistant to inhibition by the protease inhibitor aprotinin whereas salivary proteolytic activity was susceptible, which indicates differences in the specificity of proteases present in the saliva and in the gut. Digestive proteases with diverse substrate specificities could explain why genetically engineered plants expressing higher levels of PIs did not affect some populations of *Lygus* [143]. Serine and cysteine proteolytic activities were detected in the closely related plant bug, *L. lineolaris* [26]. Biochemical characterization of the *L. lineolaris* gut proteases also showed the presence of trypsin-like and chymotrypsin-like serine proteolytic activity with one gene encoding a trypsin-like protease isolated [26]. Plant bugs have strong serine proteolytic activity in the salivary gland for extra-oral digestion of plant proteins [26,144,145]. It has been postulated that these salivary gland proteases enter the insect gut with the food slurry [146,147], which may complement gut digestive proteases for efficient digestion of dietary proteins. Another hemipteran pest, the silverleaf whitefly, *Bemisia argentifolii* digests ingested plant proteins to free amino acids which are used for *de novo* protein synthesis or excreted via honeydew [148] but gut proteolytic activity was not detected. Trypsin and cathepsin B-like proteolytic activity was detected in gut extracts from the rice brown planthopper, *N. lugens* [142].

### 4.2. Insecticidal Effects of PIs against Hemiptera

Although proteolytic activity has been detected in the guts of multiple hemipteran pests, a question remains about the functional role in gut proteolytic digestion to meet nutritional requirements. In theory, these insects do not require digestion of dietary protein for development, as they feed on phloem sap which contains free amino acids at concentrations ranging from 130 mM–1050 mM [149]. Nonetheless, PIs for inhibition of aphid gut proteases have been fed in diet or expressed in transgenic plants. A cystatin from rice, OC-I, reduced population growth up to 40% and reduced fecundity in the pea aphid, the cotton aphid and the green peach aphid, when fed at levels of up to 0.25 mg/mL [138]. A barley cysteine proteinase inhibitor, cystatin was toxic to the pea aphid (LC50 of 150 µg/mL) whereas there was no significant mortality of the green peach aphid [133]. The impact of cystatin ingestion was correlated with a decrease in gut cathepsin protease activity. In contrast, cathepsins, as well as aminopeptidase activities increased in the green peach aphid after cystatin ingestion suggesting regulation of target and insensitive enzymes to overcome the effects of cystatin [133]. 

Notably, there are examples where PIs ingested by insects that lack the potential digestive target enzymes in the gut also resulted in insecticidal effects. These PIs appear to serve as defensive metabolites against phloem feeding insects but potentially by a different mechanism. The Bowman-Birk type of PI (a class of serine protease inhibitors) derived from pea seeds, exhibited insecticidal activity against the pea aphid with significant mortality [139]. Artificial cyclic peptides bearing the Bowman-Birk anti-chymotrypsin head induced the same effects on aphids indicating that the active site of this PI is responsible for the aphid toxicity. However, chymotrypsin-like proteolytic activity was not detected in aphid guts using two chromogenic chymotrypsin substrates. Chymotrypsin targeting PIs were shown to have anti-metabolic effects against three species of cereal aphid and pea aphid [137,141]. The anti-metabolic effects of PIs in the absence of the appropriate target gut protease have led to discussion of potential extra-digestive targets in these insects or an indirect PI effect on the regulation of production of digestive proteases [139,150,151]. 

As for any new approach for transgenic resistance against insect pests, appropriate assessment of the potential impact on nontarget organisms would be required prior to widespread deployment of transgenic plants in the field [152].

## 5. Other Approaches

Because of the challenges associated with management of hemipteran pests, specifically their insensitivity to Bt toxins and development of resistance to classical chemical insecticides, various additional strategies have been tested. Several alternative insecticidal molecules with Hemiptera-specific toxicity have been investigated [153]. 

A new *Chromobacterium* species isolated from insects, *Chromobacterium subtsugae* produces heat stable toxins that are highly toxic to the Southern green stink bug, *N. viridula* (L). Toxins produced by these bacteria killed 100% of the stink bug adults within six days [154]. Canatoxin (CNTX) from the seeds of *Canavalia ensiformis* (Leguminosae) are toxic to third instars of the cotton stainer bug, *Dysdercus peruvianus*, causing delayed development and eventual death [155]. This toxin was not toxic to adult insects, even at much higher concentrations, possibly due to the sensitivity of CNTX to gut proteases or to inefficient toxin activation in the adults.

Insect specific neurotoxins have also been investigated for toxicity against hemipteran pests such as the green peach aphid and the rice brown planthopper [156]. These toxins are not toxic on ingestion, but were fused to GNA, which serves as a delivery system for transport of the neurotoxin into the hemocoel [88]. GNA fused to the neurotoxic polypeptide, SF1, had significant toxicity against the rice brown planthopper in feeding assays, and reduced the survival of the green peach aphid. Avidin, derived from chicken egg white, is also toxic to the pea aphid but not to the cereal aphid [157]. The insecticidal effects of avidin are mediated by sequestering biotin, preventing absorption, causing biotin deficiency and affecting growth and survival. Pea albumin 1 (PA1b) extracted from pea seeds is toxic to several insect species, including some aphids [158,159]. PA1b is toxic to the pea aphid, toxic to the cotton aphid at high doses, but not toxic to the green peach aphid. 

Aphid myosuppressins and myosuppressin analogs designed for increased peptide stability were incorporated into the pea aphid diet, and shown to be orally toxic with dose-dependent effects. Mortality reached 100% within ten days following ingestion of the most active aphid myosuppressin, Acypi-MS [160]. These peptides may act by inhibiting contraction of visceral muscles, and stimulating enzyme secretion from digestive tissues. Stabilized mimics of pyrokinin/pheromone biosynthesis activating neuropeptides (PK/PBAN) and tachykinin-related peptides also showed toxicity against the pea aphid, possibly through disruption of digestive processes through interference with gut motility [161,162].

Plant secondary metabolites have not only been investigated for insecticidal activity but also for their impact on insect behavior. Triterpene saponins of *Quillaja saponaria* for example, exhibited strong aphicidal activity mediated by cytotoxic effects on aphid gut epithelial cells. These saponins also had strong aphid deterrent activity [163]. 

## 6. Conclusions and Future Outlook

Although hemipteran insects are becoming primary insect pests on many economically important crop plants, we have only limited understanding of their feeding biology, gut physiology and ecology. The Hemiptera appear to be unique in many aspects of their biology. As they feed on sap from phloem or xylem tissue, they encounter weaker plant defenses compared to insects that feed on the storage and reproductive tissues of the plant for example. Moreover, the targets for insecticidal molecules that are effective against other pests are unique in these insects. For example, the putative receptors for Bt toxins in these insects appear to have diverged in sequence and possibly also in structure. Knowledge of potential insecticidal targets in key hemipteran pests will be essential for the development of effective insecticidal molecules for use in transgenic plants. This knowledge could then be used to identify native insecticidal molecules or to develop mutant insecticidal molecules by directed mutagenesis.

The silencing of genes through RNA interference (RNAi) has received much attention for pest management [164,165]. While this approach has been successful against coleopteran pests [166], results for RNAi in other insect pests including the Lepidoptera [167] and the Hemiptera [167,168,169,170,171,172], have been mixed and inconsistent, and are currently inadequate for use in the management of these pests. Identification of factors limiting the efficacy of RNAi in the Hemiptera may provide for future application of this approach for management purposes. 
